# Advancements in dengue vaccines: A historical overview and prospects for following next-generation candidates

**DOI:** 10.71150/jm.2410018

**Published:** 2025-02-27

**Authors:** Kai Yan, Lingjing Mao, Jiaming Lan, Zhongdang Xiao

**Affiliations:** 1 State Key Laboratory of Bioelectronics, School of Biological Science and Medical Engineering, Southeast University, Nanjing 210096, P. R. China; 2 CAS Key Laboratory of Molecular Virology & Immunology, Shanghai Institute of Immunity and Infection Chinese Academy of Sciences, Shanghai, P. R. China; 3 University of the Chinese Academy of Sciences, Beijing, P. R. China

**Keywords:** dengue virus, mosquito-borne, vaccine, antibody-dependent enhancement

## Abstract

Dengue, caused by four serotypes of dengue viruses (DENV-1 to DENV-4), is the most prevalent and widely mosquito-borne viral disease affecting humans. Dengue virus (DENV) infection has been reported in over 100 countries, and approximately half of the world's population is now at risk. The paucity of universally licensed DENV vaccines highlights the urgent need to address this public health concern. Action and attention to antibody-dependent enhancement increase the difficulty of vaccine development. With the worsening dengue fever epidemic, Dengvaxia^®^ (CYD-TDV) and Qdenga^®^ (TAK-003) have been approved for use in specific populations in affected areas. However, these vaccines do not provide a balanced immune response to all four DENV serotypes and the vaccination cannot cover all populations. There is still a need to develop a safe, broad-spectrum, and effective vaccine to address the increasing number of dengue cases worldwide. This review provides an overview of the existing DENV vaccines, as well as potential candidates for future studies on DENV vaccine development, and discusses the challenges and possible solutions in the field.

## Introduction

Dengue is currently one of the world’s most rapidly spreading mosquito-borne diseases(Bhatt et al., 2013; WHO Guidelines Approved by the Guidelines Review Committee, 2009). Nearly half of the world’s population, residing in over 128 countries, is at risk of dengue, approximately 22,000 deaths occurring each year (Bhatt et al., 2013; Brady et al., 2012; Shepard et al., 2016). The highest incidence of dengue infection occurs in Asia, accounting for approximately 70% of the global disease burden, followed by the American tropics, the Western Pacific, and the Eastern Mediterranean regions. As of April 30, 2024, WHO reported over 7.6 million dengue cases in 2024, including 3.4 million confirmed cases and over 3,000 deaths. The threat will continue to aggravate with extended transmission seasons in endemic areas, increased vector abundance and spread, and climate change (Rocklöv et al., 2016). The rapidly expanding global footprint of dengue presents a public health challenge that remains unmet by licensed vaccines, specific therapeutic agents, or efficient vector-control strategies. To address this ongoing threat, the development of a safe and effective dengue vaccine appears to be the most promising intervention. Consequently, investments in dengue vaccine development have increased exponentially over the last decade. This review outlines the current dengue vaccines and provides perspectives on potential future directions for next-generation vaccines that show promise in combating future dengue epidemics.

### Dengue virus

**Fig. 1.**
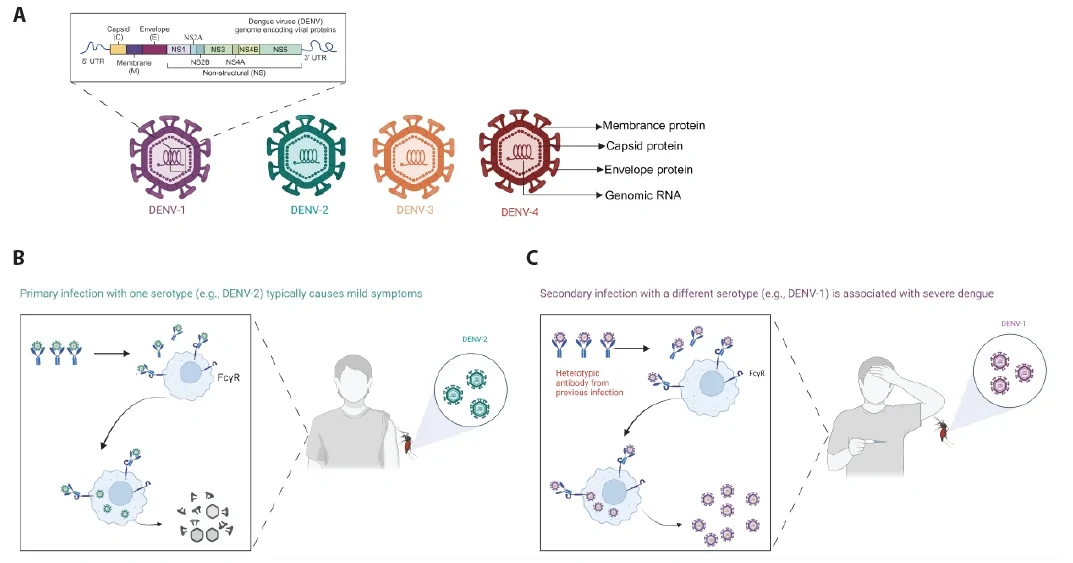
Structure of DENV and the description of ADE. (A) The structure and genome of DENV. (B, C) Mechanism of ADE in DENV Infections. ADE typically occurs during secondary infections with a different DENV serotype. (B) Following a primary infection with one serotype (e.g., DENV-2), the host produces antibodies specific to that serotype. (C) During a subsequent infection with a different serotype (e.g., DENV-1), these antibodies may fail to fully neutralize the new virus. Instead, they bind to the virus, forming antibody-virus complexes. The Fc region of these non-neutralizing antibodies interacts with Fcγ receptors on host cells, such as monocytes, macrophages, or dendritic cells. This interaction enhances the efficiency of viral entry into host cells, leading to increased viral replication. ADE not only facilitates viral dissemination but also triggers excessive inflammatory responses and immune dysregulation, further exacerbating disease severity (Created with BioRender.com).

DENV, a member of the *Flavivirus genus* within the *Flaviviridae* family, includes four genetically and antigenically distinct serotypes (DENV-1 to DENV-4) with approximately 30% divergence among them amino acid sequence (Hashem et al., 2018; Rico-Hesse, 2003; Simmons et al., 2012). A potential fifth serotype (DENV-5) has been reported but remains unconfirmed (Normile, 2013; Taylor-Robinson, 2016). DENV is an enveloped virus characterized by a positive-sense, single-stranded RNA genome of approximately 10.6 to 11.0 kb (Sabir et al., 2021).This genome encodes a single open reading frame, which is post-translationally cleaved by host and viral proteases into three structural proteins (capsid [C], pre-membrane [prM], and envelope [E]) and seven non-structural proteins (NS1, NS2A, NS2B, NS3, NS4A, NS4B, and NS5)(Kuno & Chang, 2007; Taslem Mourosi et al., 2022) as shown in Fig. 1A.

The C protein encapsulates the viral genome, which is then enclosed by a lipid bilayer membrane embedding the E and M proteins (Hasan et al., 2018). The E glycoprotein is displayed on the surface of the mature virion as a dimer and consists of three distinct domains (DI, DII, and DIII). Domain I (DI) is involved in the conformational changes necessary for viral entry and the escape of the nucleocapsid from early and late endosomes, and eventually from lysosomes with multiple hydrolases (Kang et al., 2017). Domain II (DII) contains a fusion loop that facilitates pH-dependent endosomal membrane fusion between the virus and the host cell. Domain III (DIII) is responsible for binding to cellular receptors, enabling viral entry into susceptible cells (Kanai et al., 2006; Zhang et al., 2004). Researchers have identified conserved B cell and T cell epitopes within the E protein, which make it a primary immunogen for vaccine development, despite the high mutation rate and numerous substitutions (Austin et al., 2012; Hussain et al., 2015; Lok et al., 2008). The prM protein assists in the folding and assembly of the E protein and is expressed on the surface of partially mature virions. The prM protein can also induce the production of antibodies, including those with neutralizing activity. The nonstructural (NS) proteins are primarily involved in viral replication and packaging (Falconar, 1999). Additionally, NS1 has been explored as an immunogen in vaccine candidates due to its potential to circumvent the risk of antibody-dependent enhancement (ADE)(Amorim et al., 2012, 2014; Beatty et al., 2015; Lu et al., 2013; Zheng et al., 2011).

### Dengue disease and ADE

Infection with any of the four serotypes can result in a wide spectrum of clinical manifestations, ranging from asymptomatic or mild dengue fever (DF) to potentially fatal dengue hemorrhagic fever (DHF) and dengue shock syndrome (DSS)(Guzman et al., 2016). Mortality rates of patients with DHF and DSS are approximately 2–10%. Primary infection with one serotype is believed to provide long-term and effective protective immunity against the same serotype but only transient immunity against the other serotypes (Rothman, 2004). However, a subsequent heterotypic secondary DENV infection is the greatest risk factor for developing DHF/DSS (Guzman & Harris, 2015; Sangkawibha et al., 1984). The underlying mechanism for increased disease severity is explained by ADE (Halstead et al., 1970; Halstead & O'Rourke, 1977). The ADE hypothesis suggests that, at certain concentrations, cross-reactive antibodies against the E and prM proteins of DENV bind to virions of a subsequent infecting heterologous DENV. These antibody-virus complexes are then recognized by the Fcγ receptor FcγRIIIA on target immune cells, facilitating viral entry and replication in these cells (Fig. 1B & 1C). Further study found enhanced affinity for the activating Fc receptor due to a fucosylated Fc glycans and IgG1 subclass (IgG antibodies to dengue enhanced for FcγRIIIA binding determine disease severity). This process triggers an immune cascade that induces capillary endothelial pathology and vascular leakage, potentially leading to severe dengue disease (Guzman & Harris, 2015; Rothman, 2011; Wang et al., 2017; Yacoub et al., 2015). During DENV infection, the immune response activates monocytes, macrophages (Puerta-Guardo et al., 2013), dendritic cells (Luplerdlop et al., 2006), T cells (Lühn et al., 2007), and mast cells (St John et al., 2013), which release cytokines and chemokines affecting endothelial cells etc (Avirutnan et al., 2006; Trung & Wills, 2010). This can result in cytokine storms, plasma leakage, and organ failure.

The incidence of ADE is not exactly the same between different serotypes, and the study found that the phenomenon is most likely to occur in the combination of serotypes DENV-1 and DENV-2 (Sarker et al., 2023; Shukla et al., 2020; Soo et al., 2016). ADE between these two serotypes is more common. For example, after the initial infection with DENV-2, if the second infection with DENV-1, the previously produced antibodies may not be able to effectively neutralize DENV-2, but instead enhance the infectiality of the virus through the Fc receptor-mediated pathway (Fig. 1B & 1C). The ADE phenomenon between DENV-2 and DENV-3 is also significant. Antibodies produced after DENV-2 infection may exhibit cross-reactivity in the face of DENV-3, but this reactivity is not sufficient to neutralize the virus, potentially leading to ADE. The ADE phenomenon between DENV-3 and DENV-4 should not be ignored. After an individual is infected with DENV-3, the antibodies produced before may not provide adequate protection against DENV-4, but may enhance the infectivity of DENV-4. For other viruses within the *Flaviviridae* family, there is less evidence of ADE, such as yellow fever virus, West Nile virus, Japanese encephalitis virus, and Zika virus. By now, the ADE does not occur between different members of the *Flaviviridae* family either.

## Vaccines against DENV Infection

**Fig. 2.**
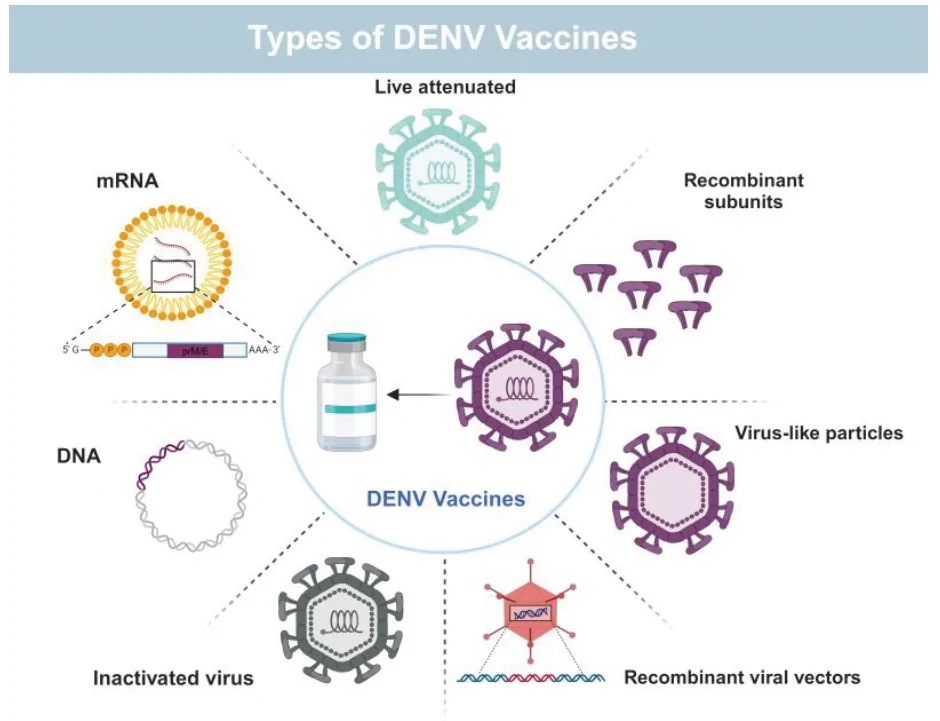
Types of DENV Vaccines. Seven types of dengue vaccines currently under investigation include live attenuated vaccines, inactivated vaccines, recombinant subunit vaccines, virus-like particle (VLP) vaccines, recombinant viral vector vaccines, and DNA/mRNA vaccines (Created with BioRender.com).

Currently, there are no specific antiviral treatments for dengue fever. The most effective strategy for preventing dengue is vaccination. Presently, two live attenuated dengue vaccines named as Dengvaxia^®^ and Qdenga^®^ have been approved for use in certain populations in dengue-endemic areas. Additionally, various other types of vaccines are in different stages of clinical trials and laboratory research (Fig. 2).

### Live attenuated vaccines

**Fig. 3.**
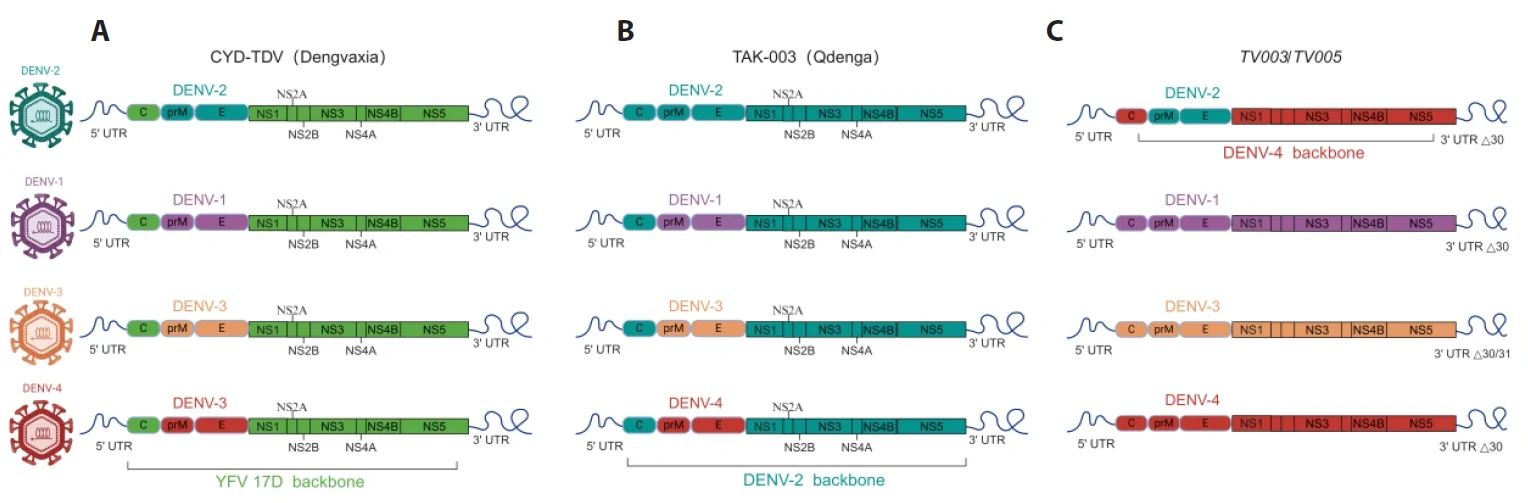
The genetic structures of the three attenuated vaccines. (A) CYD TDV is a recombinant live-attenuated tetravalent vaccine that substitutes the prM and E proteins of the 17D yellow fever vaccine virus with those of the DEN-1, -2, -3, or -4 viruses. (B) TAK-003 developed the attenuated DENV-2 strain PDK-53 through serial passaging and used it to replace the prM-E genes of DENV-1, DENV-3, and DENV-4 via genetic recombination, creating the chimeric viruses DENV2/1, DENV2/3, and DENV2/4. (c) The TV003/TV005 vaccine uses attenuated strains of DENV-1, DENV-3, and DENV-4 with a deleted portion at the 3' end, and creates a chimeric attenuated strain DENV4/2 by replacing the DENV-4 prM-E protein gene with the DENV-2 prM-E gene in the DENV-4 backbone (Created with BioRender.com).

Live attenuated vaccines are produced by reducing the virulence of the pathogen without affecting its ability to replicate. These vaccines offer strong and long-lasting immunity, as well as relatively low production costs (Bos et al., 2018; Minor, 2015). Currently, there are two approved live attenuated dengue vaccines available: Dengvaxia^®^ and Qdenga^®^, another dengue vaccine, TV003/TV005, has also completed phase III clinical trials, the genetic structures of the three attenuated vaccines were shown in Fig. 3.

**Dengvaxia^®^ :** Sanofi Pasteur has developed the yellow fever/dengue chimeric tetravalent attenuated vaccine CYD-TDV, which is the first vaccine to receive US Food and Drug Administration approval (Tully & Griffiths, 2021) and has been approved for use in several dengue-endemic countries such as Mexico, Thailand, Brazil, El Salvador, and Costa Rica (Aguiar et al., 2016). This vaccine utilizes the yellow fever virus 17D (YFV 17D) as a backbone and replaces its corresponding genes with the prM-E protein genes from DENV-1 to DENV-4 through genetic recombination shown in Fig. 2A (Guy et al., 2015). Preclinical studies have demonstrated its genetic and phenotypic stability, with no risk of transmission to mosquitoes via the oral route, and pre-existing immunity against YFV does not interfere with vaccine efficacy (Guirakhoo et al., 2006; Guy et al., 2010). Most epitopes for humoral immunity are located in the DENV E protein, whereas cellular immunity epitopes are found in the NS proteins (Pinheiro et al., 2021; Rothman, 2011; Thomas, 2023). Dengvaxia^®^ contains NS proteins from the YF-17D rather than the DENV, and while these proteins can induce cytotoxic T-lymphocyte responses and non-neutralizing antibodies, preliminary studies in monkeys indicate that these responses may not provide cross-protection (Guirakhoo et al., 2000, 2001; Tully & Griffiths, 2021). Additionally, ADE leads to increased production of the DENV's NS1 protein, an endothelial toxin that circulates through the body, causing vascular permeability and other abnormalities that contribute to severe, and sometimes fatal, dengue (Glasner et al., 2018). Therefore, the lack of neutralizing antibodies (nAb) and CD8^+^ T cell responses against the NS proteins likely contributes to the diminished protection and durability of CYD-TDV (Hou et al., 2022; Weiskopf et al., 2013).

As a tetravalent dengue vaccine, the Dengvaxia^®^ varied in its effectiveness across the four DENV serotypes with respect to virologically confirmed symptomatic dengue (Pintado Silva & Fernandez-Sesma, 2023), and have shown lower protective efficacy against DENV-2 compared to other serotypes. Results from a Phase IIb clinical trial (CYD23) conducted in Thailand (NCT00842530) showed an overall vaccine efficacy of 30.2%, with varying protection across different dengue serotypes and only 3.5% efficacy against DENV-2 (Sabchareon et al., 2012). In a Phase III trial (CYD14) conducted in healthy children in Asia (NCT01373281), the overall vaccine efficacy was 56.5%, also varying by serotype, with the lowest efficacy against DENV-2 (35.0%). The efficacy was significantly higher in children with baseline seropositivity (74.3%) compared to seronegative children (35.5%)(Capeding et al., 2014). In a Phase III trial (CYD15) conducted in children in Latin America (NCT01374516), the overall vaccine efficacy was 60.8%, with varying efficacy across the four dengue serotypes and the lowest against DENV-2 (42.3%)(Villar et al., 2015). In various clinical trials, including the Phase IIb trial CYD23 in Thailand, the Phase III trial CYD14 in Asia, and the Phase III trial CYD15 in Latin America, the vaccine showed varying degrees of protective efficacy, with lower effectiveness against DENV-2 (Capeding et al., 2014; Sabchareon et al., 2012; Villar et al., 2015). The protective efficacy was different in different age and infection background. A study on the efficacy and long-term safety of a dengue vaccine reported that, over a 25-month follow-up period, the vaccine's efficacy was 65.6% in children older than 9 years and 44.6% in those younger than 9 years. Additionally, the pooled relative risk of hospitalization for dengue was higher in children under 9 years (1.58) compared to those older than 9 years (0.5) (Hadinegoro et al., 2015; Pintado Silva & Fernandez-Sesma, 2023). Hospitalization rates were 87.6% for children aged 9 years or older and 55.9% for those younger than 9 years.

Furthermore, the ADE dengue disease occurring in seronegative sensitized by the vaccine (Halstead, 2017; Pintado Silva & Fernandez-Sesma, 2023). The long-term study of CYD-TDV in Asia and Latin America demonstrated strong protection for seropositive individuals aged 9 years and older and some protection for children aged 6–8 years. However, seronegative individuals aged 9 years and older had an increased risk of hospitalized and severe virologically confirmed dengue compared to placebo, with a similar pattern observed in seronegative participants under 9 years (Forrat et al., 2021). Additionally, based on revised efficacy and safety data for Dengvaxia^®^, it is projected that over 1,000 of the more than 800,000 children at 9-year-old vaccinated in the Philippines will be hospitalized for severe dengue, in both seronegative and seropositive individuals, despite the vaccine's overall efficacy of 69% over 4 years (Halstead et al., 2020). Therefore, The Advisory Committee on Immunization Practices (ACIP) recommends Dengvaxia^®^ vaccination for children aged 9–16 who have evidence of a previous dengue infection and reside in endemic areas. Prior to vaccination, eligible adolescents must provide evidence of past dengue infection, such as detection of anti-DENV immunoglobulin G using a highly specific serodiagnosis test or a dengue RT-PCR or NS1 antigen test (Paz-Bailey et al., 2021). Additionally, Sanofi-Pasteur is discontinuing the production of its dengue vaccine for children, citing insufficient global demand (*Dengue Vaccine Recommendations*) to sustain manufacturing.

In summary, the vaccine efficacy of Dengvaxia^®^ is influenced by the virus serotype, the age and dengue serostatus of the individual, and the host's baseline immunity to DENV (Akter et al., 2024; Guy et al., 2017). Protective efficacy is lower in children under 9 years of age and individuals with no prior exposure to dengue viruses (DENVs). Furthermore, children under 9 years of age face an increased risk of hospitalization following vaccination (Tully & Griffiths, 2021; Villar et al., 2015). The vaccine nearly doubles the risk of severe dengue disease (including DHF) in seronegative children aged 2–16 years (Kariyawasam et al., 2023; Mallapaty, 2022; Sridhar et al., 2018). Due to safety concerns, CYD-TDV is currently administered only to individuals aged 9–45 years (the specific age restrictions depend on regulatory approvals in each country) and recommended for use solely in seropositive populations as per World Health Organization (WHO) guidelines (Akter et al., 2024; Dengue vaccine: WHO position paper, September 2018 - Recommendations, 2019; Kariyawasam et al., 2023; TEAM & Immunization, 2024).

**Qdenga^®^ :** Qdenga^®^, developed by Takeda Vaccines, is the second approved tetravalent live-attenuated dengue vaccine. The vaccine acquired the DENV-2 attenuated strain PDK-53 through serial passaging and utilized it to replace the prM-E genes of DENV-1, DENV-3, and DENV-4 via genetic recombination, resulting in chimeric viruses DENV2/1, DENV2/3, and DENV2/4 shown in Fig. 2B (Osorio et al., 2011b). Preclinical studies have demonstrated the good genetic stability, safety, and immunogenicity of the vaccine (Brewoo et al., 2012; Fuchs et al., 2014; Osorio et al., 2011a). The vaccine induces a robust humoral immune response, including the production of neutralizing antibodies against DENV, cellular immune responses and anti-NS1 antibodies due to its inclusion of the non-structural genome of DENV-2. These combined responses contribute to protection against DENV infection (Brewoo et al., 2012; Henriques et al., 2013; Osorio et al., 2011b). Therefore, the immune responses generated by TAK-003 against DENV-2 capsid and non-structural proteins likely played a significant role in its high efficacy in protecting against DENV-2 (Hou et al., 2022). Furthermore, TAK-003 shows a superior efficacy profile compared to CYD-TDV, with no observed increased risk of severe disease in dengue-naive participants after vaccination. These differences are likely due to variations in the vaccine construct and the induced immune responses (Hou et al., 2022; Tricou et al., 2024).

Phase I clinical trials have shown that both high and low doses of Qdenga^®^ induce neutralizing antibody responses against all four serotypes (Osorio et al., 2014). Another Phase I trial demonstrated seroconversion rates of 84.2%, 92.1%, 86.8%, and 71.1% against the DENV-1, DENV-2, DENV-3, and DENV-4 respectively after two vaccine doses (George et al., 2015). Phase II trials (NCT01511250) confirmed the immunogenicity of the vaccine crossing all age groups and DENV serotypes (Sirivichayakul et al., 2015). Trials in children and adolescents aged 2–17 years in endemic areas (NCT02302066) showed sustained antibody responses against all serotypes up to 48 months post-vaccination (Tricou et al., 2020). Phase III trial (NCT02747927) findings indicate that TAK-003 is effective against dengue in children and adolescents in endemic countries. Follow-up results over 4.5 years demonstrate long-term efficacy and safety against all four DENV serotypes in previously exposed individuals, as well as against DENV-1 and DENV-2 in dengue-naive individuals, though efficacy against DENV-3 was not observed and the low incidence precluded evaluation against DENV-4 (Biswal et al., 2019, 2020; Tricou et al., 2024). Another Phase III trial (NCT03771963) also affirmed the tolerability and safety of TAK-003 in the latter half of its shelf life (Patel et al., 2023). Across Phase II and III trials, TAK-003 demonstrated robust immunogenicity and tolerability against all DENV serotypes, with no significant safety concerns identified during long-term follow-up post-vaccination (Biswal et al., 2019, 2020; Patel et al., 2023; Sirivichayakul et al., 2015; Tricou et al., 2020, 2024).

However, TAK-003 also faces the issue of imbalanced immunity across the four serotypes. The cumulative vaccine efficacy of two doses of TAK-003 against virologically confirmed dengue is 61.2%. Among participants who were seropositive at baseline, the cumulative efficacy is 64.2%, with effectiveness against all four dengue serotypes: 56.1% for DENV-1, 80.4% for DENV-2, 52.3% for DENV-3, and 70.6% for DENV-4. Among participants who were seronegative at baseline, the cumulative efficacy is 53.5%, with 45.4% efficacy against DENV-1, 88.1% against DENV-2, and no observed efficacy against DENV-3 and DENV-4 (Tricou et al., 2024). Furthermore, the Takeda’s recent 4–5-year Qdenga^®^ efficacy report, among 3,174 seronegative patients aged 4–16 who were vaccinated, 11 were hospitalized with DENV-3 infections, compared to 3 hospitalizations in 1,832 placebo recipients, resulting in a negative efficacy of 11.6%, similar to the Sanofi vaccine. The key difference is the lower incidence of DENV-3 cases in Takeda’s study, with DENV-4 infections also being rare (Halstead, 2024). Takeda plans a large post-authorization effectiveness study to evaluate TAK-003's impact on severe/hospitalized dengue cases, particularly due to DENV-3 and DENV-4, addressing limitations from the pivotal licensure study (mondiale de la Santé & Organization, 2024). Overall, TAK-003 is the second dengue vaccine to be WHO-prequalified, recommended for vaccination of children aged 6–16 years in high dengue burden areas, administered in two doses three months apart (mondiale de la Santé & Organization, 2024; Patel et al., 2023; WHO, 15 May 2024).

**TV003/TV005 :** The National Institute of Allergy and Infectious Diseases (NIAID) developed the dengue fever vaccine TV003/TV005 by deleting a portion of the 3' untranslated region (3′ UTR) of the virus shown in Fig. 3C, aiming to prevent the four serotypes of DENVs. This vaccine uses attenuated strains obtained by deleting a portion of the 3' end of the DENV-1, DENV-3, and DENV-4 viruses. Additionally, using the attenuated DENV-4 strain as a backbone, the gene encoding the prM-E protein from DENV-2 replaces the corresponding gene of the DENV-4 backbone to obtain the chimeric attenuated strain DENV4/2 (Whitehead, 2016). TV003 contains a mixture of genetically modified viruses from the four DENV serotypes with 10^3^ ± 0.5 plaque-forming units (PFU), while TV005 increases the dosage of DENV-2 (Kirkpatrick et al., 2015; Precioso et al., 2015).

Phase I clinical trials (NCT01072786) showed that a single dose of TV003 induced a tetravalent or higher neutralizing antibody response in 90% of participants (Durbin et al., 2013). Clinical trials of TV005 (NCT01072786, NCT01436422) showed that three months after a single dose of TV005, it elicited a tetravalent response in 90% and a trivalent response in 98% of vaccinees. Compared to TV003, the higher dose of DENV-2 component in the TV005 trial increased the observed frequency of DENV-2 immunogenicity (Kirkpatrick et al., 2015). Butantan-DV, a freeze-dried tetravalent attenuated live dengue fever vaccine developed by Butantan Institute, similar to TV003, showed neutralizing antibody responses to all four serotypes in phase II clinical trials conducted in Brazil, with no severe adverse events (Kallas et al., 2020). Use of the genetically modified, low-virulence rDEN2Δ30 virus in the TV003 vaccine resulted in no viremia, rash, or neutropenia in subjects six months after vaccination, demonstrating the vaccine's strong protective efficacy (Kirkpatrick et al., 2016). Results from a Phase III double-blind trial (NCT02406729) conducted in Brazil showed that the overall vaccine efficacy over two years was 79.6%, 73.6% in participants with no prior evidence of dengue exposure, and 89.2% in participants with a history of dengue exposure. Efficacy was 80.1% in participants aged 2–6 years, 77.8% in those aged 7–17 years, and 90.0% in those aged 18–59 years. The efficacy against DENV-1 was 89.5% and against DENV-2 was 69.6%. No cases of DENV-3 and DENV-4 were detected during the follow-up period (Kallás et al., 2024).

Unlike the use of a single backbone vector in CYD-TDV and TAK-003 formulations, TV003/TV005 achieves balanced immunogenicity against all four dengue serotypes by introducing nucleotide deletions in the 3′ UTR of DENV-1, DENV-3, and DENV-4, which plays a critical role in viral RNA replication, along with other mutations in the non-structural proteins (Alvarez et al., 2005; Deng et al., 2020; Men et al., 1996). As a result, TV003/TV005 differs significantly from CYD-TDV and TAK-003 in terms of viral particle structure, infectivity and immunogenicity (Deng et al., 2020; Whitehead, 2016). Additionally, because TV003/TV005 includes the viral backbone from DENV-1, DENV-3, and DENV-4, which can stimulate T-cell immune responses in the body, this vaccine may offer enhanced immunogenicity and protective efficacy (Prompetchara et al., 2020). Actual research results also indicate that after vaccination, the vaccine induces not only neutralizing antibody responses but also CD8^+^ T-cell responses (Kirkpatrick et al., 2015; Lindow et al., 2013; Weiskopf et al., 2015).

Live attenuated vaccines have demonstrated strong potential in preventing dengue fever, showing effective control, particularly when applied in specific populations and regions, significantly reducing both the incidence and severity of the disease. However, different vaccines vary in their protective efficacy against each serotype of the virus. According to current research data, vaccine effectiveness also depends on individual baseline immune status and age. Future vaccine development and improvement will continue to focus on enhancing protection and safety against all DENV serotypes.

### Inactivated vaccines

Inactivated vaccines are known for their high safety, rapid development, and ability to induce immune responses against multiple antigens simultaneously. However, inactivated vaccines only express the part of the DENV genome responsible for structural proteins, preventing them from stimulating an immune response against NS proteins. As a result, they have weaker immunogenicity, a limited ability to induce cellular immune responses, and carry a risk of ADE (Akter et al., 2024). In the development of inactivated vaccines, recent advancements have primarily focused on improvements in virus inactivation techniques, the application of novel adjuvants, and the combined use of other types of vaccines. Four dengue-inactivated vaccines are currently in clinical trial phases. Clinical trial results of the monovalent DENV-1 PIV candidate vaccine formulated with alum adjuvant (NCT01502735) showed favorable tolerability and acceptable immunogenicity in dengue-naive healthy adults, capable of eliciting humoral immune responses and triggering CD4^+^ T-cell responses (Friberg et al., 2020; Martinez et al., 2015). Phase I clinical trials conducted in the United States with a tetravalent formalin-inactivated vaccine formulated with alum (TDEN-PIV) (NCT01666652) demonstrated induction of high and balanced neutralizing antibody responses against all four dengue serotypes at one-month post-immunization, with no severe adverse reactions observed up to 12 months (Schmidt et al., 2017). Additionally, in clinical studies conducted among dengue-seropositive healthy adults (NCT01702857), a composite adjuvant (AS01E and AS03B) formulated inactivated vaccine induced high and balanced neutralizing antibody responses against all four DENV serotypes at one month post-second immunization, showing sustained immunogenicity for up to 12 months and good tolerability with no safety concerns observed within three years post-vaccination (Diaz et al., 2018, 2020). Recent research indicates that a tetravalent dengue inactivated-vaccine (alum/AS01E/AS03B) can induce B-cell and T-cell responses lasting up to 12 months in adults (Friberg et al., 2022). Furthermore, results from a phase I/II clinical trial (NCT02421367) showed that the DPIV for-mulated with AS03B adjuvant using a 0–1–6-month dosing schedule induced higher peak neutralizing antibody responses at one-month post-final immunization compared to 0–3 month and 0–1-month schedules. However, similar levels of memory B cells and neutralizing antibodies were observed at 12 months between the 0–1–6- and 0–3-month schedules, suggesting the superiority of the 0–3 month and 0–1–6-month schedules over the 0–1-month schedule (Lin et al., 2020a).

In the development of inactivated dengue vaccines, purified formalin-inactivated (PIV) DENV-2 (S16803) induced high titers of neutralizing antibodies and demonstrated robust immunogenicity and protective efficacy in mouse and rhesus monkey models (Putnak et al., 1996a, 1996b). Additionally, the safety and immunogenicity of DENV-2 PIV, recombinant subunit (DENV-2-R80E), and attenuated vaccines (DENV-2 PDK-50) were assessed concurrently, showing that these candidate vaccines could all induce high titers of neutralizing antibodies and provide partial protection against DENV-2 challenge, with DENV-2 PDK-50 displaying the most stable antibody response (Putnak et al., 2005). Furthermore, DENV-2 vaccines inactivated with aminoethyl benzene trioxane (AMT) preserved the binding capacity of specific monoclonal antibodies and induced T-cell responses similar to those induced by live viruses (Raviprakash et al., 2013). Reports indicated that psoralen-inactivated DENV serotype 1 (DENV-1 PsIV) demonstrated immunogenicity in mice and Aotus nancymaae monkeys, and compared to formalin inactivation, a tetravalent vaccine (PsIV) inactivated with psoralen-inactivated methods could better induce neutralizing antibody responses (Maves et al., 2010, 2011; Sundaram et al., 2020). In mice, PsIV vaccines formulated with Advax-PEI and Advax-2 adjuvants induced higher levels of neutralizing antibodies compared to aluminum hydroxide (alum), with only the Advax-2 adjuvant group eliciting a superior IFN-γ response (Wu et al., 2022).

These study results indicated that in the development of dengue vaccines, inactivated vaccines remained important candidate strategies. Their immunogenicity and long-term protective effects could be improved by utilizing advanced adjuvants and combining them with other types of vaccines, such as DNA vaccines and live attenuated vaccines, offering new strategies for vaccine development.

### Recombinant subunit vaccines

Recombinant subunit vaccines had advantages such as minimal side effects, good biological safety, and ease of large-scale production. However, it had relatively weak immunogenicity, requiring adjuvants and involving complex immunization procedures. The list of Recombinant subunits DENV vaccine candidates are given in Table 2.

Expression Systems Drosophila S2 cells and CHO are all uses to acquire the recombinant protein vaccines against DENV. Among them, V180 was the most advanced in clinical trials. Currently, Merck's V180 recombinant subunit vaccine has entered Phase I clinical trials. The vaccine contained truncated proteins with 80% of the N-terminal E protein of DENV1-4, produced in S-2 insect cells, and mixed with ISCOMATRIX™ adjuvant (aluminum hydroxide), inducing high levels of neutralizing antibodies against all four DENV serotypes in rhesus monkeys and demonstrating protection against viremia following DENV1-4 virus challenge (Govindarajan et al., 2015). Data from two clinical trials (NCT00936429 and NCT01477580) demonstrated that Alhydrogel-adjuvanted formulations of V180 were generally well tolerated and induced a modest immune response in Flavivirus-naive individuals (Manoff et al., 2015, 2019) Additionally, V180 formulations were well tolerated and could increase serum neutralization titers and the frequency of DENV seropositivity in participants previously vaccinated with dengue LATV (TV003 or TV005)(Durbin et al., 2020).

Studies indicated that fusion proteins of DENV-2 EDⅡ/EDⅢ or EDⅡ/EDⅢ/NS1 expressed in insect cells could elicit neutralizing antibodies in mice, although P28 did not significantly enhance neutralizing antibody levels (García-Machorro et al., 2013). Furthermore, recombinant DENV-DIII proteins expressed in insect cells, either individually or as a tetravalent combination, could also induce serotype-specific neutralizing antibodies in mice (Block et al., 2010), whereas NS1 vaccines based on S-2 insect cell expression could prevent lethal vascular leakage syndrome caused by virulent DENV and significantly protect mice from fatal challenge by DENV-2 when vaccinated with NS1 from DENV1-4 (Beatty et al., 2015).

The DENV-4 EDIII protein expressed in prokaryotes, combined with various adjuvants (FCA, Montanide, ISA720, Alum), produced high levels of neutralizing antibodies and cellular immune responses in mice (Babu et al., 2008). Recombinant DENV-2 EDIII protein induced neutralizing antibodies in mice and protected neonatal mice from lethal challenge (Zhang et al., 2007). Tandem proteins of the four DENV serotype EDIII domains expressed in *E. coli* induced specific antibodies in mice (Chen et al., 2007b). Fusion of Neisseria meningitidis carrier protein P64K with DENV-4 EDIII protein provided partial protection in mice (Lazo et al., 2009). Combined expression of EDIII from DENV-1 and DENV-2 with P64K induced neutralizing antibodies against different genotypes of DENV-1 and DENV-2 in monkeys (Bernardo et al., 2008, 2009; Hermida et al., 2004a, 2004b, 2006). The tetravalent EDIII-P64K recombinant protein combined with alum adjuvant-induced neutralizing antibodies and cellular immunity in mice, providing partial protection against DENV infection (Lazo et al., 2014). The tetravalent vaccine composed of fusion proteins from DENV-1, -3, and -4 EDIII domains with P64K and DENV-2 EDIII and C proteins demonstrated protective immune responses in mice (Izquierdo et al., 2014). A recombinant vaccine formulated with EDIII and C proteins from all four DENV serotypes induced functional immune responses in both mice and monkeys (Suzarte et al., 2014, 2015). Another subunit vaccine composed of prokaryotically expressed DENV C protein, aggregated with ODN 39M to form nucleocapsid-like particles, induced IFN γ cellular responses in both mouse and monkey models without generating virus-specific antibodies, potentially avoiding the risk of ADE (Gil et al., 2016). A novel dengue vaccine candidate composed of the consensus sequence of domain III of the DENV1-4 E protein (cEDIII), expressed in *E. coli*, produced neutralizing antibodies against all dengue serotypes in BALB/c mice but only induced neutralizing antibodies against DENV-2 in monkey models (Chen et al., 2013; Leng et al., 2009).

Recombinant nonstructural DENV proteins were also a significant focus of research; deletion or mutation of cross-reactive epitopes on the NS1 protein may yield safer and more effective vaccines (Sun et al., 2017). The NS3 and NS5 proteins, expressed in *E. coli*, were investigated as subunit vaccines. The NS3 protein stimulated the production of anti-NS3 IgG antibodies and TH1 cellular responses in mice, while the NS5 protein induced high levels of specific antibodies and provided partial protection against viral challenge (Alves et al., 2016; Ramírez et al., 2014). The combination of recombinant modified NS1 (NS1 with C-terminal cross-reactive epitopes replaced by Japanese encephalitis virus NS1 region) and NS3 proteins led to enhanced immune responses and improved protection in mouse models (Kao et al., 2019).

The major shortcomings of the *E. coli* expression system include the lack of post-translational modifications, uncertainty in protein refolding, and endotoxin issues. Alternative expression systems can potentially avoid these problems. For instance, a recombinant subunit vaccine containing the consensus sequences of the EDIII domains from all four dengue serotypes expressed in *Saccharomyces cerevisiae* generated specific antibodies and a balanced immune response in mice (Nguyen et al., 2013). The DENV-2 EDIII and tetravalent EDIII subunit vaccines, expressed in *Pichia pastoris*, induced neutralizing antibodies in mice (Batra et al., 2010; Etemad et al., 2008). Additionally, the LTB-scEDIII fusion protein, expressed in *Saccharomyces cerevisiae* and comprising the B subunit of the heat-labile enterotoxin from *E. coli* (LTB) and the consensus dengue envelope domain III from all four serotypes (scEDIII), stimulated systemic and mucosal immune responses in mice when administered orally, either as whole recombinant yeast cells (WC) or as cell-free extracts (CFE). Notably, the oral administration of CFE elicited higher humoral and cellular immune responses as well as higher neutralizing titers (Bal et al., 2018).

To address the poor immunogenicity and need for adjuvants and multiple doses in subunit vaccines, lipidated consensus domain III proteins from all four dengue serotypes (LcED III) were developed. A single dose of LcED III, without any additional adjuvant, was sufficient to induce neutralizing antibodies against all four dengue serotypes in mice (Chiang et al., 2011). In the baculovirus expression system, recombinant proteins (cE80(D4) and cE80(max)) containing the extracellular domain of the dengue envelope protein, based on the 3127 strain, elicited specific antibodies and Th2-biased cellular immune responses against all four DENV serotypes (Sun et al., 2017). In a rice expression system, a single polypeptide chain (tEDIII-Co1) containing the EDIII domains from all four dengue serotypes and coenzyme 1 (Co1) induced strong antigen-specific B and T cell responses in a mouse model (Kim & Kim, 2019). Transient expression of the consensus domain III of the E proteins from all four dengue serotypes, fused with cholera toxin B subunit (CTB-cEDIII), in *Nicotiana benthamiana*, led to the induction of systemic and mucosal immune responses against DENV following oral immunization in mice (Huy & Kim, 2017). Research on dengue recombinant protein subunit vaccines in mammalian expression systems remains limited. However, previous studies have constructed a polymeric immunoglobulin scaffold (PIG) to enhance the uptake of the candidate vaccine by immune cells. The fusion protein cEDIII-PIGS, containing the DENV consensus E domain III (cEDIII) and the PIG scaffold, expressed in Chinese hamster ovary (CHO) cells and transgenic plants, induced high titers of IgG antibodies and showed neutralizing potential against DENV-2 in mice without the need for adjuvants (Kim et al., 2017).

Recombinant subunit vaccines for DENV have shown promising results in preclinical and clinical trials, demonstrating potential for inducing protective immune responses with varying degrees of efficacy. Advances in expression systems and adjuvants have improved vaccine development, though challenges such as immunogenicity and stability remain. Future efforts should focus on enhancing vaccine efficacy, achieving broad protection against all DENV serotypes. Continued optimization of vaccine formulations and adjuvants, coupled with advancements in expression systems, will likely enhance immunogenicity and broaden protection.

### Virus-like particles (VLPs) vaccines

Virus-like particles (VLPs) vaccines is identical or similar to that of the native virus and exhibit antigenic epitopes akin to natural viruses due to their three-dimensional structure (Nooraei et al., 2021). This enables them to efficiently induce both specific humoral and cellular immune responses. However, the applicability of VLP vaccines is limited, as only a few viruses can self-assemble into VLPs. Additionally, similar to other vaccines, VLP vaccines require the use of adjuvants and multiple injections to complete the immunization regimen. The list of VLPs DENV vaccine candidates is given in Table 3.[Table t4-jm-2410018]

Sugrue et al. (1997) first expressed the DENV-1 C-prM-E structural proteins in yeast, producing immunogenic DENV-like particles capable of eliciting neutralizing antibodies in rabbits. Similarly, recombinant DENV-1 VLPs composed of prM and E proteins expressed in *P. pastoris* induced virus-neutralizing antibodies and cellular immune responses in immunized mice (Tang et al., 2012). Recent studies have shown that recombinant extracellular domains of E proteins from DENV-1, DENV-2, DENV-3, and DENV-4 expressed in yeast, even without prM protein, self-assemble into immunogenic VLPs capable of inducing serotype-specific neutralizing antibodies in mice (Khetarpal et al., 2017; Mani et al., 2013; Poddar et al., 2016; Tripathi et al., 2015). A bivalent VLP (mE1E2bv VLP), composed of DENV-1 and DENV-2 E proteins co-expressed and assembled in *P. pastoris*, retained serotype-specific antigenic integrity. It predominantly elicited EDIII-focused virus-neutralizing antibodies in BALB/c mice without demonstrating significant ADE (Shukla et al., 2017). A chimeric antigen HBcAg-EDIII-2, where DENV-2 EDIII is fused into the c/e1 loop exposed on the surface of HBcAg antigen, expressed in both *Escherichia coli* and yeast, formed VLPs with immunogenicity in mice, inducing specific neutralizing antibodies against DENV-2 (Arora et al., 2012, 2013). Using the yeast *P. pastoris*, co-expression of all four DENV serotypes E proteins assembled into a tetravalent VLPs (T-mVLP) without prM protein. T-mVLP preserved antigenic integrity and immunogenicity against all four serotypes following a three-dose immunization regimen in mice, inducing EDIII-directed antibodies capable of neutralizing all four DENV serotypes without enhancing sublethal DENV-2 infection in AG129 mice sensitive to dengue fever (Rajpoot et al., 2018). Additionally, a fusion of the EDIII regions of all four DENV serotypes with the HBV surface S protein, co-expressed with non-fused S antigen, formed DSV4 VLPs. These induced effective and durable neutralizing antibodies against all four serotypes in mice and monkeys, protecting mice from the DENV-4 challenge (Ramasamy et al., 2018). Furthermore, immunization with a recombinant tetravalent DENV1-4 VLPs vaccine expressed in yeast induced specific antibodies against all DENV1-4 antigens in mice, with higher antibody titers compared to monovalent VLPs vaccines (Liu et al., 2014).

In the silkworm expression system, recombinant dengue sVLPs containing the full-length C-prM-E proteins of DENV-1 and DENV-4 were produced, inducing humoral immune responses in mouse models (Utomo et al., 2022). Additionally, DENV-2 VLPs vaccines generated in mosquito cells with enhanced cleavage of prM-E induced high levels of neutralizing antibodies in mice and enhanced neutralizing antibody and EDIII-specific binding antibody responses in *Macaca fascicularis* following attenuated live virus challenge (Suphatrakul et al., 2015). Recombinant tetravalent DENV VLP vaccines expressed in mammalian 293T cells induced specific humoral and cellular immune responses in mice (Zhang et al., 2011). VLPs resembling DENV-2 particles, composed of structural proteins C-prM-E and NS2B/NS3 protease complex, produced at lower temperatures, elicited the highest titers of neutralizing antibodies in mice (Boigard et al., 2018; Fan et al., 2024). A novel tetravalent VLPs vaccine (DENV1-4 VLPs), co-expressing prM and E proteins of all four DENV serotypes in FreeStyle 293F cells, exhibited high neutralizing activity against all serotypes without ADE, surpassing immunity induced by DNA vaccination (Urakami et al., 2017). Furthermore, this VLP vaccine induced long-lasting neutralizing antibody responses against all four DENV serotypes in non-human primates (NHPs) for up to a year without detectable ADE activity, which effectively reduced viral replication in rodents and NHPs (Thoresen et al., 2024). VLPs produced in *Nicotiana benthamiana* expressing DENV-1 structural protein (C-prM-E) and NS5 induced humoral immune responses in mice (Ponndorf et al., 2021). A VLP vaccine expressing DENV-2 EDIII and hepatitis B virus surface S protein (HBsAg) using measles virus (MV) vectors elicited robust neutralizing responses against MV, HBV, and DENV-2 in MV-sensitive mice (HuCD46Ge-IFNarko)(Harahap-Carrillo et al., 2015).

DENV VLP vaccines have shown strong potential in various expression systems and animal models, demonstrating effective neutralizing antibodies against multiple DENV serotypes. Future work should focus on enhancing immunogenicity, improving stability, exploring new expression systems and immunization regimens, and validating efficacy and safety through clinical trials.

### Recombinant viral vector vaccines

**Fig. 4.**
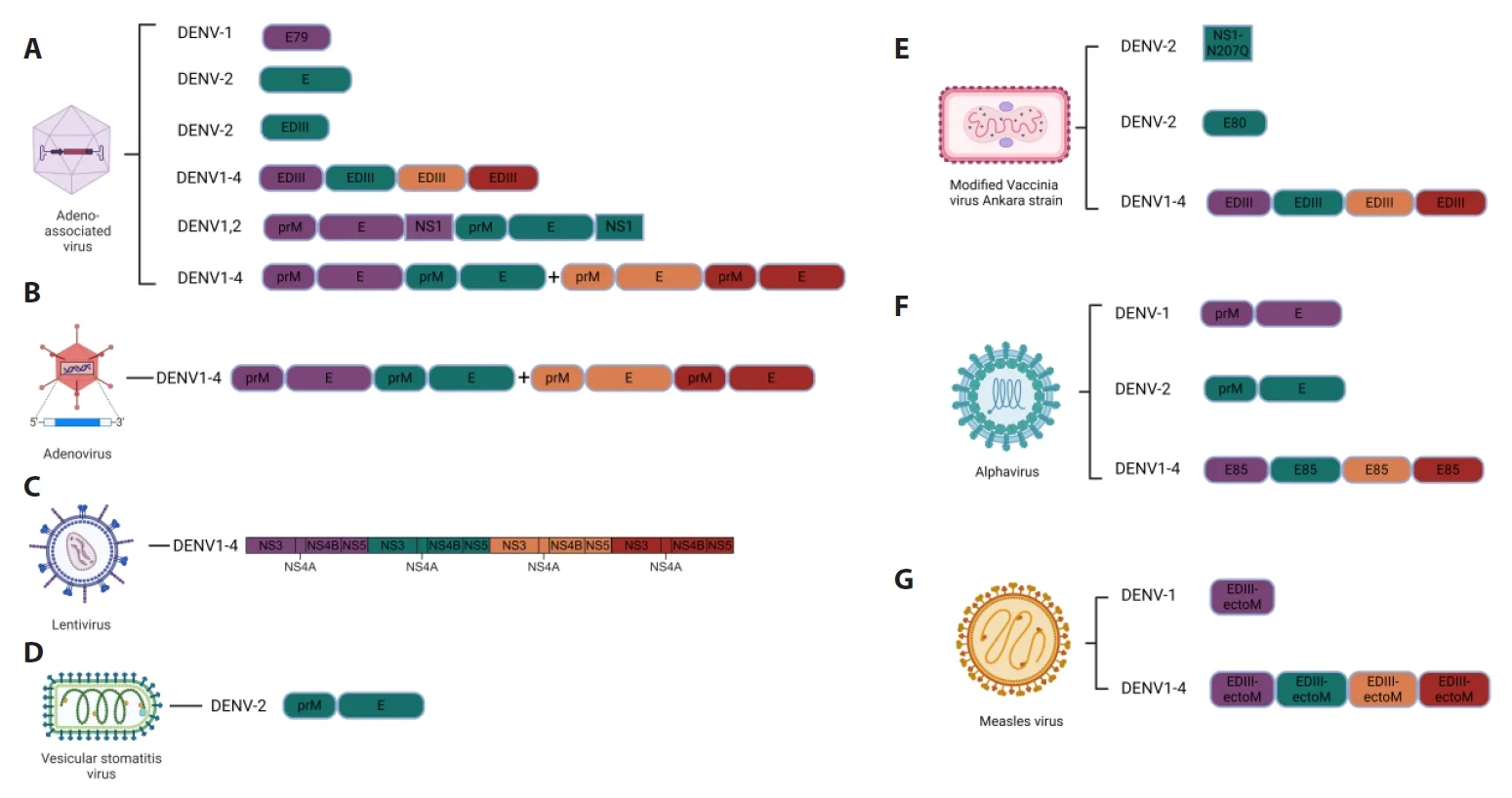
Structural Diagram of Dengue Recombinant Viral Vaccines. Currently, various viral vectors are utilized in vaccine development, including (A) AAV, (B) AdV, (C) Lentiviral vector, (D) VSV, (E) Modified vaccina virus vector (Ankara strain), (F) Alphavirus vector, (G) MV, and others (Created with BioRender.com).

Although no viral vector vaccines progressed to clinical trail stage, various viral vectors are utilized in DENV vaccine development shown in Fig. 4, including adenovirus (AdV), adeno-associated virus (AAV), lentiviral, measles virus (MV), modified vaccinia virus Ankara strain (MVA), alphavirus, vesicular stomatitis virus (VSV)(Tomczyk & Orzechowska, 2013) and others (Lundstrom, 2020). Among them avoiding the pre-immunity by rare serotype of adenovirus and abrogating of integration of viral genes into the host genome by alphaviruses with single strain RNA genomes showed great potential prospect.

Initial studies using a replication-defective recombinant human adenovirus type 5 (Ad5) expressing the N-terminal 80% or DIII of the E protein [amino acid (aa) residues 1-395] of DENV-2 could be used in a mouse model to elicit DENV-2 specific neutralizing antibodies and a Th1-biased cell response. Additionally, the immune response induced by the rAd-EDIII prime/ pVAX-EDIII boost regimens appeared to be relatively more potent (Jaiswal et al., 2003; Khanam et al., 2006). In another study, the recombinant adenovirus vaccine rAdV5 expressing DENV1-4 EDIII induced cell-mediated immune responses and virus-neutralizing antibodies specific to each of the four DENVs in mice. This suggested that pre-existing immunity to AdV5 might facilitate the uptake of rAdV5 vectored vaccines into antigen-presenting cells (Khanam et al., 2009). A cAdVaxD vaccines vector expressing prM and E protein of DENV1/2 and DENV3/4, respectively (CAdVax-Den12 and CAdVax-Den34) induced humoral and cellular immune responses against all four DENV serotypes in mice (Holman et al., 2007). In Rhesus Monkeys, intramuscular injection of rCAdVax-Den12 and CAdVax-Den34 generated rapid, long-term and high-titer antibodies capable of neutralizing all four DENV serotypes, offering significant protection against viremia of all four DENV serotypes (Raviprakash et al., 2008).

AAV and lentiviral vectors are also commonly used in vaccine development. Research has shown that an AAV-based vaccine encoding DENV-1 truncated E protein (79E,C-terminal truncated E) induced humoral immune responses in mice (Li et al., 2012). A tetravalent lentiviral T-cell vaccine (LV-DEN) targeting immunogenic regions of DENV non-structural proteins (NS3, NS5, NS4B, and NS4A) exhibited potent CD8^+^ T-cell immunogenicity and provided significant protection against all four DENV serotypes in mouse models (Nemirov et al., 2023). MV were also widely used in vaccine research. An MV vector encoding a minimal combined dengue antigen composed of the EDIII fused to the ectodomain of the membrane protein (ectoM) from DENV1-4 induced long-term neutralizing response against all four serotypes in MV-susceptible mice without cross-reactivity to other serotypes. The ectoM component played an adjuvant role, which was crucial for enhancing the immunogenicity of EDIII (Brandler et al., 2007, 2010).

MVA was another extensively used viral vaccine vector. An MVA expressing truncated DENV-2 E protein induced high levels of DENV-2 neutralizing antibodies in mice and completely protected rhesus monkeys from DENV-2 infection after three immunizations (Men et al., 2000). An MVA expressing the DENV1-4 EDIII induced humoral responses against all four DENV serotypes in immunized mice, with neutralizing activity against DENV-2 (Mintaev et al., 2023). Additionally, an MVA virus vector vaccine based on DENV-2 NS1 (rMVA-D2-NS1-N207Q) induced robust NS1-specific antibody and T-cell responses in mice (Wilken et al., 2023).

Lauretti et al. (2016) constructed a VSV-based candidate vaccine expressing DENV-2 prM and E proteins, which induced neutralizing antibodies against DENV-2 and protected animals from lethal DENV infection.

Alphavirus is an enveloped, single-stranded positive-sense RNA virus and also a commonly used viral vector. Studies have demonstrated that in the Venezuelan equine encephalitis virus (VEEV) replicon particle (VRP) system, a candidate vaccine expressing DENV-1 prM and E (D1ME-VRP) induced high levels of DENV-1-specific IgG and neutralizing antibodies in *Macaca fascicularis* when administered via a heterologous DNA prime-VRP boost regimen using two doses of DNA vaccine and a third dose of VRP vaccine, resulting in completely protecting against viral viremia (Chen et al., 2007a). A VRP vaccine encoding DENV-2 prM and E proteins vaccination by a prime-boost regimen enhanced neutralizing antibody levels and protected 3-week-old weanling BALB/c mice from the lethal DENV-2 challenge (White et al., 2007). A non-propagating vaccine vector based on VRP expressing configurations of DENV soluble E dimers (E85,a C-terminally truncated soluble form of E that represents 85% of the protein) induced tetravalent neutralizing antibody responses in macaques, providing protective responses against all four serotypes, with these responses being distinct from those induced by live virus infection (White et al., 2013). Similarly, immunization of neonatal mice with a tetravalent VRP vaccine expressing DENV1-4 E85 proteins elicited robust neutralizing antibodies and T-cell immune responses, conferring protection against lethal challenges in neonatal mice after a single immunization (Khalil et al., 2014).

Viral vector vaccines have shown significant efficacy in the development of DENV vaccines, inducing strong humoral and cellular immune responses and providing protection against multiple DENV serotypes. In the future, the selection and modification of viral vectors hold promise for playing a crucial role in DENV prevention and control.

### Nucleic acid vaccines

**Fig. 5.**
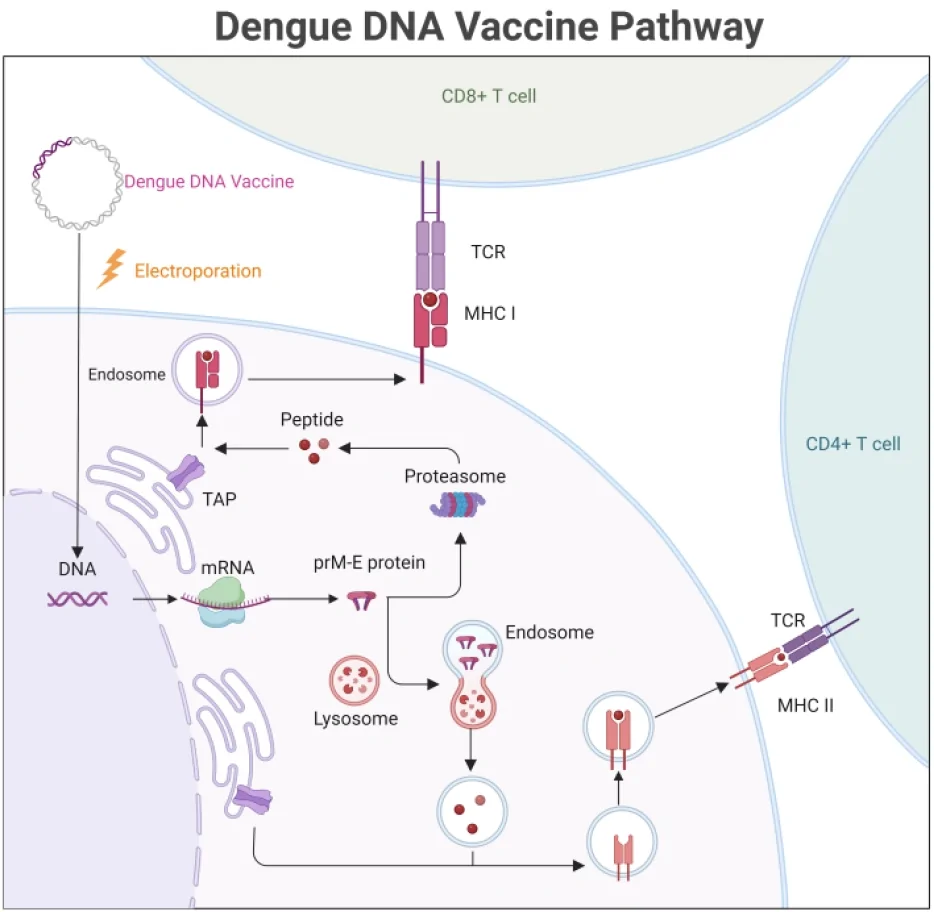
Dengue DNA Vaccine Pathway. Dengue DNA vaccines carry genetic sequences encoding DENV antigens, such as E protein, prM protein, or NS1 protein, typically delivered in plasmid form. The vaccine is introduced into host cells through methods like electroporation, intramuscular injection, or gene gun delivery. Once inside the host cells, the DNA utilizes the host's transcription and translation machinery to express DENV antigen proteins. These antigen proteins are presented by major histocompatibility complex (MHC) class II molecules to helper T cells (CD4+ T cells), which activate B cells to differentiate into plasma cells and produce neutralizing antibodies. Simultaneously, antigen proteins are presented by MHC class I molecules to cytotoxic T cells (CD8+ T cells), which, upon activation, kill virus-infected cells, thereby reducing viral replication and spread (Created with BioRender.com).

Nucleic acid vaccines, representing third-generation vaccine technology, include DNA and mRNA vaccines, presenting antigens in their natural form, inducing robust humoral and cellular immune responses (Fig. 5).

**DNA vaccine :** Both monovalent plasmid DNA vaccine (D1ME100) and tetravalent dengue DNA vaccine (TVDV) finished the phase I clinical trial. The D1ME100 expressing DENV-1 prM and E genes showed significant dengue-specific T cell IFN-γ responses, but its immunogenicity was poor, failing to elicit strong neutralizing antibody responses (Beckett et al., 2011). However, a tetravalent dengue DNA vaccine (TVDV) formulated with the Vaxfectin^®^ adjuvant significantly enhanced neutralizing antibody responses in rabbits and NHPs (Porter et al., 2012; Raviprakash et al., 2012). Unfortunately, in a phase I clinical trial (NCT01502358), TVDV also showed moderate cellular immune responses but did not induce satisfactory neutralizing antibody responses in subjects (Danko et al., 2018).

One reason for the low antibody responses generated by DNA vaccines is their relatively low transfection efficiency (Manoj et al., 2004). Recently, electroporation (EP) technology has been demonstrated to significantly enhance DNA delivery efficiency, thereby increasing antigen expression levels and the intensity of immune responses (Khan, 2013). DNA vaccines expressing DENV-2 prM/E, when delivered via EP, have been shown to protect mice from lethal DENV-2 infection and to induce effective DENV-2-specific antibody responses in rabbits (Chen et al., 2016; Zheng et al., 2017). Compared to traditional intramuscular (i.m.) injection, EP immunization with DNA vaccines expressing DENV-1 and DENV-4 prM-E antigens induced durable humoral and cellular immune responses, providing effective protection to mice against lethal DENV1/4 challenge (Sheng et al., 2019). Additionally, a bivalent vaccine delivered by EP, comprising DENV-1 prM/E and DENV-2 prM-E, elicited neutralizing antibodies against DENV1 and DENV2 and conferred protection to mice from lethal challenge (Zheng et al., 2017). An EP-delivered DENV-3 prM-E DNA vaccine induced robust neutralizing antibody responses and antigen-specific T-cell responses, offering comprehensive protection against lethal infections of DENV-1, DENV-3, and DENV-4, and partial protection against DENV-2 (Feng et al., 2020). Furthermore, recent studies have indicated that using EP and/or intradermal (i.d.) delivery routes enhances the immunogenicity and protective efficacy of tetravalent dengue DNA vaccines in NHPs compared to i.m. alone (Williams et al., 2019).

Two DNA candidate vaccines expressing either DENV-3 or DENV-4 prM and E proteins elicited specific neutralizing antibodies after three immunizations and conferred 80% protection against the lethal DENV-3 challenge in mice (De Paula et al., 2008; Lima et al., 2011). Two vaccine candidates were constructed by upstream insertion of the DENV-2 and DENV-3 prM genes into the DENV-2 E gene, named pCID2EtD2prM and pCID2EtD3prM, respectively. Both vaccines induced DENV-2 specific neutralizing antibodies and cellular immune responses, showing varied protective efficacy against lethal DENV-2 challenge, with pCID2EtD3prM providing the highest protection at 90% (Dias et al., 2021).

The NS1 protein was able to induce strong humoral and cellular immune responses and was often developed in combination with prM/E proteins to enhance vaccine efficacy (Chen et al., 2016; Khan, 2013; Zheng et al., 2017). A DNA vaccine expressing DENV-1 prM-E-NS1 antigen alone (prM-E-NS1) or co-expressing with GM-CSF (a DNA vaccine adjuvant) (pCAG-DV1-GM) resulted in a long-term IgG response, high levels of IFN γ and IL-2, strong CTLs activity, and sufficient protection in DENV-1-challenged mice. Results also showed that GM-CSF did not significantly enhance the immune response, but the immune responses induced by these two vaccines seemed stronger than those induced by expressing viral prM-E alone (Zheng et al., 2011). Additionally, a DNA candidate vaccine co-expression DENV-2 prM-E-NS1 and GM-CSF induced neutralizing antibodies and partially protected mice from the DENV-2 challenge (Lu et al., 2013). A DNA vaccine encoding DENV-2 E-NS1 proteins induced strong humoral and cellular immune responses against E and NS1 proteins, providing complete protection to BALB/c mice from the DENV-2 challenge (Pinto et al., 2022).

It is expected that the immunogenicity of DNA vaccines in large animals can be effectively improved via new delivery methods such as Ep or microneedles, which step the DNA vaccines to clinical.

**Fig. 6.**
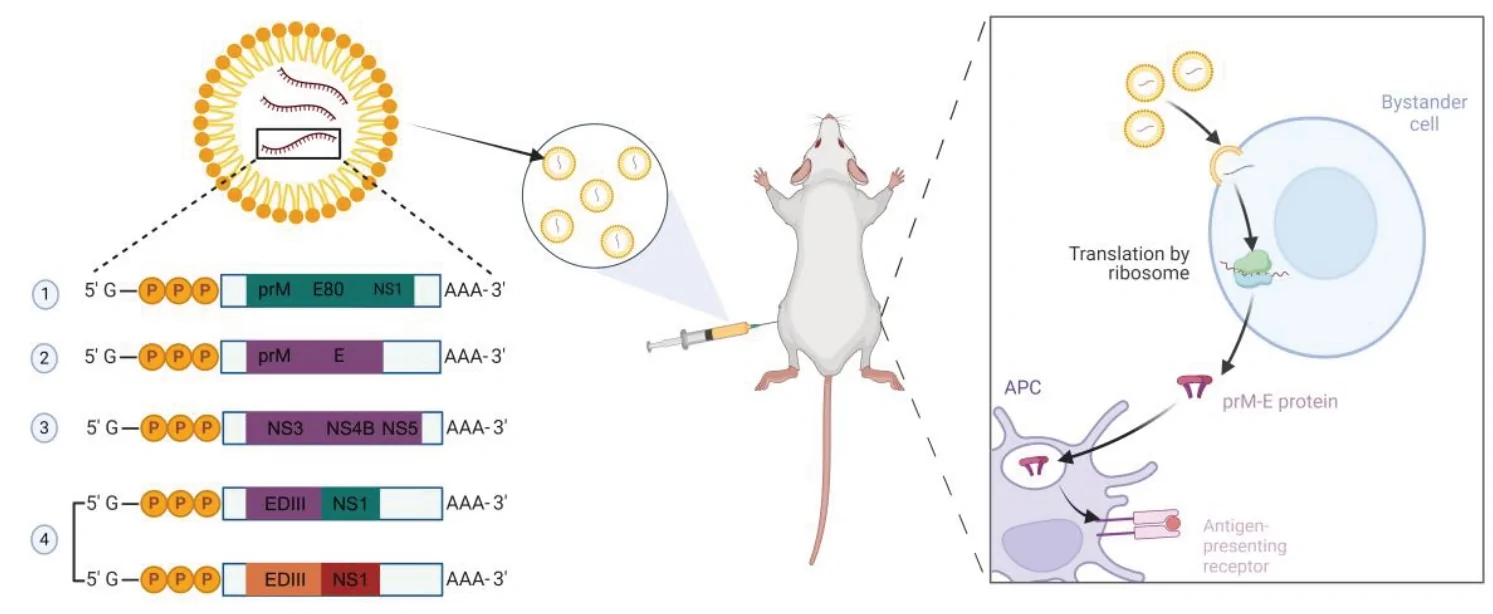
Diagram of the Structure and Mechanism of Dengue mRNA Vaccines. The dengue mRNA vaccine leverages host cells to express DENV antigens, inducing a rapid and specific immune response against the virus and demonstrating significant potential for developing safe and effective vaccines. The vaccine contains mRNA molecules encoding DENV antigens, such as the E protein, prM protein, or NS1 protein, which are typically encapsulated in lipid nanoparticles (LNPs) to protect the mRNA from degradation and enhance cellular uptake. Upon administration, LNPs are taken up by host cells, releasing the mRNA into the cytoplasm, where it is translated into DENV antigens by the host’s ribosomes. The synthesized antigen proteins are either processed within host cells or secreted extracellularly, where they are captured and broken down by antigen-presenting cells (e.g., dendritic cells), thereby triggering both humoral and cellular immune responses to effectively prevent DENV infection (Created with BioRender.com).

**mRNA vaccine :** mRNA vaccines, as innovative vaccines, have a simple and rapid preparation process, are easily scalable and can induce potent humoral and cellular immune responses, which has proven advantageous in combating the COVID-19 pandemic (Fig. 6)(Fang et al., 2022). Zhang et al. developed DENV non-replicable mRNA vaccines based on prM-E, E80 and NS1 of DENV-2, delivered using lipid nanoparticles (LNPs). The results demonstrated that both E80-mRNA alone or the combination of E80-mRNA and NS1-mRNA induced high levels of specific neutralizing antibodies and antigen-specific T-cell immune responses, providing complete protection against lethal DENV-2 challenge in BALB/c mice (Zhang et al., 2020). However, the DENV-2 E80 mRNA vaccine also induced serotype cross-reactive immune resulted in ADE. Wollner et al. (2021) used an LNP-encapsulated non-replicable mRNA vaccine encoding DENV-1 prM and E proteins, which induced strong humoral and cellular immune responses in C57BL/6J mice after i.m. injection and provided protection against lethal DENV-1 challenge in interferon-deficient AG129 mice, significantly reducing DENV2-induced ADE. Claude Roth et al. (2019) used a non-replicable mRNA vaccine encoding DENV-1-NS, composed of conserved and highly antigenic epitopes from NS3, NS4B, and NS5 regions, which induced strong cellular immunity and provided significant protection against DENV-1 infection in HLA Class I transgenic mice without eliciting neutralizing antibodies. He et al. (2022) developed a modified non-replicable mRNA vaccine containing the EDIII and NS1 encapsulated in LNP. This multi-target vaccine induced a robust antiviral immune response and increased neutralizing antibody titers that blocked all four DENV serotypes in vitro without significant ADE. However, more rational antigen design for enhanced cross-protection and elimination ADE is also a challenge for mRNA DEN vaccines. In addition, the long-term immune response induced by mRNA vaccine and the evaluation of safety, immunogenicity and efficiency in large animal models or clinical trails are also the effort that need to be focused.

Despite challenges related to immunogenicity and storage stability, nucleic acid vaccines, including DNA and mRNA vaccines have demonstrated effective protection in animal models and clinical trials, and are expected to play a crucial role in future vaccine development.

## Conclusions and Future Directions

**Fig. 7.**
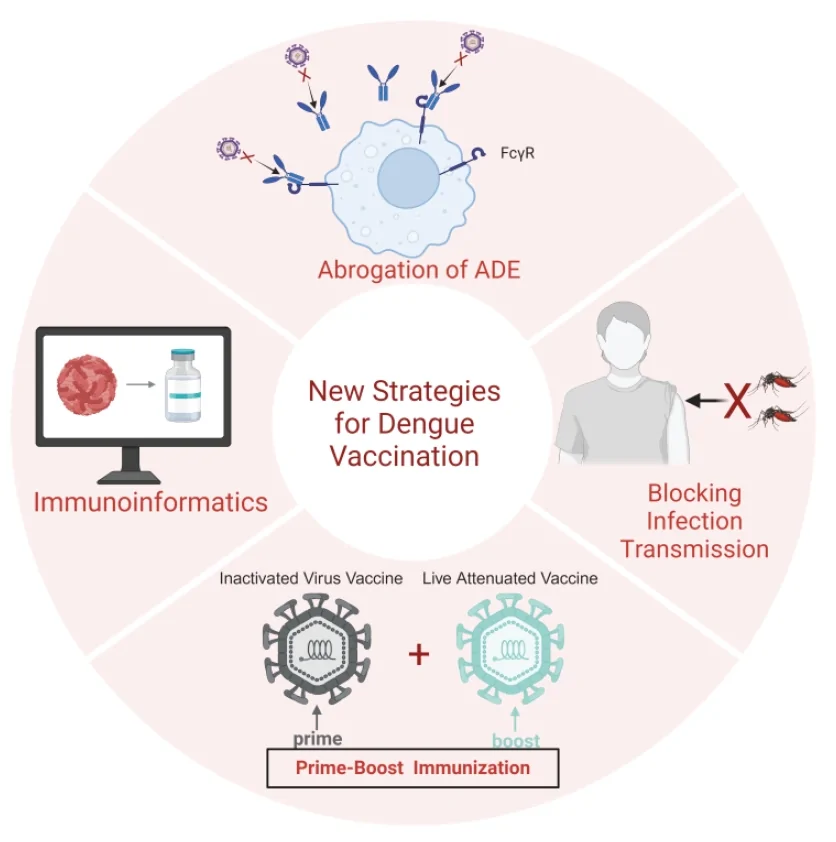
New Strategies for Dengue Vaccination. The innovative strategies for dengue vaccine development include (1) Abrogation of ADE: Strategies to mitigate the Fcγ receptor (FcγR)-mediated ADE effect, preventing enhanced viral infection; (2) Blocking Infection Transmission: Strategies to prevent the transmission of DENV from mosquitoes to humans; (3) Immunoinformatics: Leveraging computational tools to design effective vaccine candidates by optimizing antigen selection and predicting immune responses; (4) Prime-Boost Regimen: A sequential immunization approach using an inactivated virus vaccine as a primer and a live attenuated vaccine as a booster to elicit robust and durable immune responses (Created with BioRender.com).

To effectively address the global dengue burden, a multifaceted approach combining vector control, enhanced vaccine development, and novel immunization strategies is crucial. Current vaccines, such as Dengvaxia^®^ and Qdenga^®^, face limitations in providing balanced and durable protection against all four DENV serotypes, especially in seronegative populations. Overcoming these challenges requires innovative strategies, including precise antigen targeting to avoid ADE, optimizing immune responses to all serotypes, ensuring long-term efficacy, and addressing accessibility and side-effect concerns. Advances in prime-boost regimens, immunoinformatics-driven vaccine designs, and transmission-blocking vaccines offer promising avenues (Fig. 7). Additionally, next-generation nucleic acid vaccines, such as mRNA and circular RNA platforms, hold the potential to revolutionize dengue prevention by offering single-dose, broad-spectrum, and ADE-free solutions. This comprehensive understanding underscores the need for integrated approaches to support the development of safer, more effective dengue vaccines.

### Antigen strategies for DENV vaccines

Current dengue control strategies focus primarily on vector control, but this alone may not be sufficient to control dengue epidemics in the long term. Vector control and vaccination have the potential to be mutually reinforcing and may have synergistic effects in preventing dengue fever. The complexity of dengue pathology is not limited to ADE. Based on previous clinical data, the development of a dengue vaccine that is effective in both dengue-naïve and previously exposed individuals and that protects against all four serotypes remains a major challenge. Though two dengue fever vaccines, Dengvaxia^®^ and Qdenga^®^, have been approved for human vaccination in specific groups of people in dengue fever epidemic areas. However, both vaccines fail to provide a balanced immune response to DENV1-4. Dengvaxia^®^ exhibits less than 50% efficacy against DENV-2, while Qdenga^®^, although offering higher overall protection than Dengvaxia^®^, lacks efficacy against DENV-3 in seronegative participants (Tricou et al., 2024). A safe, broad-spectrum and effective vaccine still needs to be developed to deal with the growing number of dengue cases worldwide.

To achieve this goal, the following five challenges are concluded. i) Abrogation of ADE, Vaccine design needs to avoid the ADE, which is an immune response that occurs when non-neutralizing antibodies enhance the infectivity of the virus. ii) Balance of immune response: Vaccines need to stimulate an equivalent protection against all four DENV serotypes to avoid an imbalance in immune response. iii) Persistence of vaccine efficacy: there is a need to ensure that the protection provided by the vaccine is long-term, especially in DENV endemic areas. iv) Vaccine accessibility: Vaccines need to be viable in terms of cost, production and distribution in order to be used in resource-limited countries. v) Side effects of vaccines: Possible side effects of vaccines need to be monitored and managed, especially during mass vaccination.

Among all the challenges, the abrogation of ADE mainly includes the following is strategies. i) Select those that can induce the production of antibodies against specific viral epitopes that have high neutralizing power and are not susceptible to cross-reaction with other viruses. For example, in vaccine design against DENV, antibodies targeting DENV envelope protein domain III (EDIII) are selected because they are highly type-specific and less likely to cause ADE. ii) Designing vaccines to induce specific types of immune responses: By designing vaccines to induce primarily type-specific immune responses, reducing the production of cross-reactive antibodies that cause ADE. For example, through vaccine design, it is possible to induce an antibody response that targets primarily EDIII, rather than antibodies that target the precursor membrane protein (prM) and fused ring epitope (FLE), which are more likely to cross-react and cause ADE. iii) Avoid or minimize exposure to immunodominant epitopes: In vaccine design, avoid or minimize exposure to those immunodominant epitopes that can be recognized by cross-reactive antibodies, such as prM and FLE, which not only have poor neutralization ability, but may also promote viral replication and disease severity through the Fcγ receptor-mediated pathway. iv) Use of mutated or modified antigens: By mutating or modifying viral antigens, the occurrence of ADE can be reduced. For example, eliminating FLE by introducing mutations, while still demonstrating effective neutralizing epitopes, can reduce the occurrence of ADE. v) Use of non-structural proteins as antigens: Some studies have proposed the use of non-structural proteins (such as NS1) as antigens that can induce a protective immune response without the risk of inducing ADE. vi) Optimize vaccine formulation and adjuvants: By using novel adjuvants and optimizing vaccine formulation, it is possible to enhance vaccine immunogenicity while reducing the risk of ADE. For example, RBD-Fc combined with novel adjuvants could serve as a highly effective, broad-spectrum vaccine against future viral infections and potentially avoid ADE.

The goal of these strategies is to improve the safety and efficacy of vaccines by precisely regulating the immune response and stimulating the production of effective neutralizing antibodies, while avoiding the production of non-neutralizing or hyponeutralizing antibodies that may contribute to ADE.

### Prime-boost strategies

Studies demonstrated that in a prime-boost vaccination regimen using three tetravalent dengue vaccines, including DNA vaccine (TDNA), inactivated vaccine (TPIV), and live attenuated vaccine (TLAV), the TPIV/TLAV prime-boost regimen induced the highest and most sustained neutralizing antibody responses in rhesus monkeys, providing complete protection against all four DENV serotypes (Simmons et al., 2010). The study also indicated that TDNA and TLAV vaccines effectively induced T-cell responses, while the TPIV vaccine promoted memory B-cell responses. TLAV vaccine significantly enhanced T-cell responses to TPIV vaccine, which may contribute to sustaining the prolonged antibody response of TPIV/TLAV vaccines (Sun et al., 2021). Results from a phase I heterologous prime-boost study clinical trial (NCT02239614) also showed that compared to TDEN-LAV/TDEN-PIV, TDEN-PIV/TDEN-LAV produced higher tetravalent seroconversion rates of neutralizing antibodies (Lin et al., 2020a).

### Advances in immunoinformatics revolutionize dengue vaccine design

The development of immunoinformatics has improved and expanded dengue vaccine design strategies. In a study, Sitara Nasar et al. (2024) utilized bioinformatics tools to identify conserved regions of the DENV NS1 protein containing epitopes capable of eliciting balanced immunity against all four serotypes, while excluding conserved epitopes known to produce cross-reactive antibodies against cellular machinery, and experimentally validated the immunogenic potential of the resulting N- and C-terminal deleted NS1 variant (dNS1), proposing it as a substitute for NS1 in future vaccine formulations. Basheer et al. (2023) designed a multi-epitope tetravalent vaccine targeting DENV1-4 serotypes using conserved sequences from EDIII, prM, and NS1 proteins through an immunoinformatic approach, demonstrating high immunogenicity, non-allergenicity, and non-toxicity, with immune simulations suggesting its ability to elicit both antibody and cell-mediated responses, while molecular docking and MD simulation validated strong binding affinity and stability in the vaccine-TLR3 complex, highlighting its potential to induce a balanced im-mune response against all dengue serotypes without adverse effects. Fadaka et al. (2021) used computational approaches to design multi-epitope vaccines against DENV, with immune simulation analysis indicating that DV-1 could elicit a specific immune response, while codon optimization and in silico cloning confirmed its expression in *E. coli*, and molecular dynamics revealed its stability with minimal RMSF against TLR4. A study analyzing NS1, prM, and DEIII from DENV1-4 serotypes of Pakistani isolates utilized machine learning and reverse vaccinology approaches to develop an mRNA vaccine, with codon optimization of the conserved sequence to enhance translation efficiency, identification of T-cell and B-cell epitopes from the consensus protein, and generating 3D structures, thereby laying the groundwork for future research into the development of effective vaccines to prevent dengue infection (Mukhtar et al., 2022). Additionally, Ali et al. (2017) used immunoinformatics to develop a multi-epitope subunit dengue vaccine designed to elicit diverse immune responses, incorporating predicted B-cell, TC, and TH cell epitopes, along with β-defensin as an adjuvant at the construct's N-terminal. The vaccine showed potential to induce humoral and cell-mediated immunity, was non-allergenic, and displayed strong antigenicity. Structural modeling and molecular docking with TLR-3 confirmed effective ligand-receptor interactions, while in silico cloning verified its expression and translation efficiency.

### Strategies focused on blocking infection transmission

The vast global populations of Aedes aegypti, which sustain the circulation of all four DENV serotypes, underscore the urgent need for vaccines to control dengue transmission (Halstead, 2024). The WHO has warned that half of the global population is at risk of contracting the mosquito-borne DENV (Donmez, 21.07.2023 - Update : 22.07.2023). 11 years ago, in 2013, the Aedes albopictus mosquito was established in 8 EU/EEA countries, affecting 114 regions. By 2023, it has spread to 13 countries and 337 regions (*Increasing risk of mosquito-borne diseases in EU/EEA following spread of Aedes species*, 22 Jun 2023). Additionally, different variants of DENV are found worldwide, with transmission facilitated by increased human travel and urbanization. The movement of people between regions and countries spreads diverse DENV strains, making outbreak control and prevention more challenging. Furthermore, climate change and environmental factors can affect mosquito vector distribution, further extending the global reach of DENV transmission (Sabir et al., 2021). Moreover, the mosquito vectors of dengue transmit other important viruses, including yellow fever, chikungunya and Zika viruses.

The release of Wolbachia-carrying mosquitoes in parts of Yogyakarta in 2016 led to a 77% reduction in dengue rates in those areas compared to untreated areas over the subsequent years, showcasing a potential strategy to eliminate dengue (Callaway, 2020). Additionally, transmission-blocking vaccines represent a novel approach in vaccine design, aimed at preventing vectors like mosquitoes from becoming infected when they take a blood meal from vaccinated vertebrate hosts, thereby reducing virus transmission. Unlike traditional vaccines, these vaccines target key molecules involved in the virus's development within mosquitoes or molecules expressed by the mosquitoes that facilitate viral infection. By inducing antibodies in the host, these vaccines block the virus's transmission from the infected host to the mosquito, thereby protecting other animals from infection (Londono-Renteria et al., 2016). Their advantages include the ability to interrupt viral transmission at an early stage, offering a sustainable and eco-friendly solution that maintains ecological balance. Immunizing people in high-risk areas and those traveling to these regions can effectively prevent the spread of the virus to lower-risk areas.

Vaccination against dengue should be part of a comprehensive strategy to control the disease, which also includes vector control, proper case management, community education, and engagement. Effective dengue control programs must continue to prioritize comprehensive vector control as a crucial element (TEAM & Immunization, 2024).

Currently, various vaccine candidates for dengue are at preclinical and clinical trial stages of development based on different approaches. As a new generation of nucleic acid vaccines, the approval of multiple viral mRNA vaccines provides favourable support for the development of DENV mRNA vaccines. Development of replicative mRNA and circular RNA vaccines will contribute the effective DENV vaccines. For example, the use of circular RNA technology to encode specific antigens, such as EDIII and NS1, can explore a new vaccine strategy that can prevent viral infection with a single dose without the risk of ADE. Overall, an in-depth understanding of the mechanism of DHF and ADE, to provide new ideas and a theoretical basis for the development of the next generation of DENV vaccines.

## Figures and Tables

**Table 1.** Inactivated-based DENV vaccine candidates involving the clinical trial phase

Serotype	Inactivation method	Adjuvant	Animal models/Status	Ref
DENV-1	Formalin	Alum	Phase I: NCT01502735	Friberg et al. (2020), Martinez et al. (2015)
DENV1-4	Formalin	Alum, AS01_E, _AS03_B_	Phase I:NCT01666652,NCT01702857	Diaz et al. (2018, 2020), Friberg et al. (2022), Schmidt et al. (2017)
DENV1-4	Formalin	AS03_B_	Phase I/II: NCT02421367	Lin et al. (2020b)
DENV1-4	Formalin	Alum	Phase I: NCT02239614	Lin et al. (2020a)

**Table 2.** Recombinant subunit-based DENV vaccine candidates detected in NHP or clinical trials

Serotype	Antigen	Expression system	Adjuvant	Animal models/Status	Ref
DENV1-4	E80	Drosophila S2 cells	ISCOMATRIX™ adjuvant	Indian rhesus macaques	Govindarajan et al. (2015)
DENV-1	E80	Drosophila S2 cells	Alhydrogel™	Phase I: NCT00936429	Manoff et al. (2015)
DENV1-4	E80	Drosophila S2 cells	ISCOMATRIX™, Alhydrogel™	Phase I: NCT01477580	Manoff et al. (2019)
DENV1-4	E80	Drosophila S2 cells	Alhydrogel™	Phase I: NCT02450838	Durbin et al. (2020)
DENV-1	DIII-P64k	*E. coli*	Freund's adjuvant	*Macaca fascicularis*, Rhesus monkeys	Bernardo et al. (2008)
DENV-2	DIII-P64k	*E. coli*	Freund's adjuvant	*Macaca fascicularis*	Hermida et al. (2006)
DENV1-4	DIII-C	*E. coli*	Aluminum hydroxide	American green monkeys	Suzarte et al. (2014, 2015)
DENV2	DIII-C+ ODNs (m2216, poly IC and 39M)	*E. coli*	Aluminum hydroxide	Vervet monkeys	Gil et al. (2015)
DENV1-4	cEDIII	*E. coli*	Aluminum hydroxide	*Macaca cyclopis*	Chen et al. (2013)

**Table 3.** VLP-based DENV vaccine candidates

Serotype	Antigen	Expression system	Animal models	Ref
DENV-1	C-prM-E	*P. pastoris*	Rabbits	Sugrue et al. (1997)
DENV-1	prM-E	*P. pastoris*	BALB/c mice	Tang et al. (2012)
DENV1,2,3,4	E ectodomain	*P. pastoris*	BALB/c mice	Khetarpal et al. (2017), Mani et al. (2013), Poddar et al. (2016), Tripathi et al. (2015)
DENV1-2	E	*P. pastoris*	BALB/c and AG129 mice	Shukla et al. (2017)
DENV-2	HBcAg-EDIII-2	*E. coli*	BALB/c mice	Arora et al. (2012)
DENV-2	HBcAg-EDIII-2	*P. pastoris*	BALB/c mice	Arora et al. (2013)
DENV1-4	E	*P. pastoris*	BALB/c and AG129 mice	Rajpoot et al. (2018)
DENV1-4	EDIII-HBV S	*P. pastoris*	BALB/c, AG129, C57BL-6, C3H, Macaques	Ramasamy et al. (2018)
DENV1-4	prM-E	*P. pastoris*	BALB/c mice	Liu et al. (2014)
DENV1-4	prM-E_F108A_	FreeStyle 293F cells	BALB/c mice,NIH Swiss outbred mice	Urakami et al. (2017)
DENV1-4	prM-E_F108A_	FreeStyle 293F cells	Cynomolgus monkeys,Common marmosets,AG129 mice	Thoresen et al. (2024)
DENV1-4	C-prM-E	silkworm	BALB/c mice	Utomo et al. (2022)
DENV-2	prM-E	Mosquito cells	BALB/c mice,Cynomolgus macaques	Suphatrakul et al. (2015)
DENV1-4	prM-E	293T	BALB/c mice	Zhang et al. (2011)
DENV-2	C-prM-E+NS2B-NS3	Expi293TM cells	BALB/c mice	Boigard et al. (2018)
DENV-1	C-prM-E+ΔNS5 NSP	*Nicotiana benthamiana*	BALB/c mice	Ponndorf et al. (2021)

**Table 4. t4-jm-2410018:** DNA-based DENV vaccine candidates

Serotype	Antigen	Delivery mode	Animal models/Status	Ref
DENV1-4	prM-E	EP,i.d.	*Macaca fascicularis* monkeys	Williams et al. (2019)
DENV-1	prM-E		Phase I: NCT00290147	Beckett et al. (2011)
DENV1-4	prM-E+Vaxfectin^®^	i.m.	Indian rhesus monkeys	Porter et al. (2012)
DENV1-4	prM-E+Vaxfectin^®^		Phase I: NCT01502358	Danko et al. (2018)

E.P., electroporation; i.m., intramuscular; i.d., intradermal
